# Supplementary material for: Non-rapid eye movement sleep and wake neurophysiology in schizophrenia
Source: eLife. 2022 May 17;11:e76211. doi: 10.7554/eLife.76211 (PMC9113745; doi:10.7554/eLife.76211)
Supplement: Supplementary file 1. [file elife-76211-supp1.docx]

**Supplemental file 1**

**Non-rapid eye movement sleep and wake neurophysiology in schizophrenia**

**Authors:** Nataliia Kozhemiako^1†^, Jun Wang^2†^, Chenguang Jiang^2†^, Lei A. Wang^3^, Guan-chen Gai^2^, Kai Zou^2^, Zhe Wang^2^, Xiao-man Yu^2^, Lin Zhou^3^, Shen Li^4^, Zhenglin Guo^3^, Robert G. Law^1^, James Coleman^3^, Dimitrios Mylonas^5^, Lu Shen^7^, Guoqiang Wang^2^, Shuping Tan^6^, Shengying Qin^7^, Hailiang Huang^3,8^, Michael Murphy^4^, Robert Stickgold^9,10^, Dara S. Manoach^5^, Zhenhe Zhou^2•^, Wei Zhu^2•^, Mei-Hua Hall^4•^, Shaun M. Purcell^1,10•*^ & Jen Q. Pan^3•*^

**Affiliations:**

1. Department of Psychiatry, Brigham and Women’s Hospital, Harvard Medical School; Boston, USA
2. The Affiliated Wuxi Mental Health Center of Nanjing Medical University; Wuxi, China
3. Stanley Center for Psychiatric Research, Broad Institute of MIT and Harvard; Boston, USA
4. Department of Psychiatry, McLean Hospital, Harvard Medical School; Boston, USA
5. Department of Psychiatry, Massachusetts General Hospital, Harvard Medical School; Boston, USA
6. Huilong Guan Hospital, Beijing University; Beijing China
7. Bio-X Institutes, Shanghai Jiao Tong University; Shanghai China
8. ATGU, MGH, Harvard Medical School; Boston, USA
9. Beth Israel Deaconess Medical Center; Boston, USA
10. Department of Psychiatry, Harvard Medical School; Boston, USA

^†^ - co-first authors; • - co-senior authors

* - corresponding authors (Jen Q. Pan, jpan@broadinstitute.org ; Shaun M. Purcell, smpurcell@bwh.harvard.edu)

***Supplementary file 1: Group differences in EEG parameters between SCZ and CTR***

| **Sleep microstructure metrics** | **SCZ**  **Mean ± SD(min, max)** | **CTR**  **Mean ± SD(min, max)** | **Number of channels with p_adj_<0.05** | **Significant p_adj_ range** | **Effect size across channels with p_adj_<0.05** |
| --- | --- | --- | --- | --- | --- |
| ***SS density, n/min*** | 1.3±0.49 (0.7–2.1) | 1.5±0.47 (0.9-2.2) | 20 | 3e-04 - 0.0117 | -0.79 - -0.6 |
| ***SS amplitude, uV*** | 24.4±6.73 (16.9–33.3) | 28.6±6.73 (20-37.5) | 49 | 3e-04 - 0.0467 | -0.82 - -0.43 |
| ***SS ISA, a.u.*** | 1.3±0.24 (1.2–1.5) | 1.5±0.2 (1.3-1.7) | 34 | 3e-04 - 0.048 | -0.96 - -0.63 |
| SS duration, s | 0.9±0.09 (0.8–0.9) | 0.9±0.07 (0.8-0.9) | 0 |  |  |
| SS chirp, a.u. | -0.23±0.151 (-0.31–-0.14) | -0.25±0.117 (-0.34--0.15) | 0 |  |  |
| SS frequency, Hz | 11.1±0.41 (10.9–11.2) | 11.2±0.48 (11-11.4) | 0 |  |  |
| **FS density, n/min** | 1.7±0.82 (1–2.6) | 2.4±0.8 (1.4-3.3) | 53 | 3e-04 - 0.0443 | -1.27 - -0.56 |
| **FS amplitude, uV** | 17.2±4.62 (11.8–23) | 19.5±5.3 (12.4-27.1) | 22 | 0.0013 - 0.0457 | -0.62 - -0.41 |
| **FS ISA, a.u.** | 1.3±0.18 (1.2–1.4) | 1.4±0.15 (1.3-1.5) | 1 | 0.0373 | -0.73 |
| **FS duration, s** | 0.8±0.09 (0.7–0.8) | 0.8±0.07 (0.8-0.9) | 35 | 0.0057 - 0.0477 | -0.98 - -0.69 |
| **FS chirp, a.u.** | -0.22±0.109 (-0.27–-0.16) | -0.17±0.101 (-0.25--0.09) | 20 | 0.0027 - 0.0437 | -1.09 - -0.59 |
| FS frequency, Hz | 13.8±0.37 (13.6–13.9) | 13.7±0.37 (13.4-13.8) | 0 |  |  |
| **SO density, n/min** | 13.3±2.16 (11.9–14.6) | 12.3±1.68 (10.1-13.7) | 30 | 3e-04 - 0.0483 | 0.59 - 1.16 |
| **SO duration, s** | 1.1±0.22 (0.9–1.3) | 1±0.14 (0.8-1.1) | 36 | 3e-04 - 0.033 | 0.66 - 1.92 |
| **SO slope, a.u.** | 171.1±42.04 (107.9–249.2) | 208.3±48.59 (122.7-323.2) | 44 | 3e-04 - 0.0377 | -1.14 - -0.55 |
| SO neg peak amplitude, uV | 36.2±8.37 (25.5–49.2) | 38.5±8.7 (25-54.4) | 0 |  |  |
| SO peak-to-peak amplitude, uV | 61.3±13.96 (43.9–80.9) | 64±13.8 (42.2-88.9) | 0 |  |  |
| SS SO coupling strength, z | 2.4±2.37 (0.8–4.5) | 2.8±2.34 (1.2-4.6) | 0 |  |  |
| **SS SO coupling overlap, z** | 2.1±1.08 (1.2–2.8) | 2.7±0.91 (1.7-3.4) | 13 | 0.0063 - 0.046 | -1.14 - -0.62 |
| **SO phase angle when SS occur,°** | 352.5±70.01 (326.7–374.9) | 373.8±56.91 (354.5-389.9) | 2 | 3e-04 - 0.019 | -1.07 - -1 |
| FS SO coupling strength, z | 6.7±4.06 (3.8–9.5) | 5.8±2.97 (3.6-8) | 0 |  |  |
| FS SO coupling overlap, z | 2.9±1.26 (2.2–3.4) | 2.9±1.03 (1.9-3.5) | 0 |  |  |
| **SO phase angle when FS occur,°** | 233±22.52 (218.8–241.7) | 240.8±26.8 (220.6-252.2) | 2 | 0.045 - 0.0497 | -0.65 - -0.41 |
| **MMN Amplitude, uV** | 1.3±0.98 (2–0.6) | 1.6±0.99 (2.6-0.6) | 1 | 0.012 | -0.7 |
| MMN Latency, s | 180.7±25.65 (179–183.8) | 182.3±26.65 (178.7-186.2) | 0 |  |  |
| **P50 S2/S1 ratio** | 0.8±0.4 (0.7–1) | 0.6±0.39 (0.4-0.8) | 10 | 0.001 - 0.044 | 0.62 - 1.06 |
| P50 S1 amplitude, uV | 2.1±1.04 (1.6–2.9) | 2.2±1.04 (1.5-3.1) | 0 |  |  |
| P50 S2 amplitude, uV | 1.5±0.82 (1.2–1.8) | 1.2±0.72 (0.9-1.5) | 0 |  |  |
| **ASSR Power, dB** | -0.7±0.42 (-1–-0.4) | -0.5±0.43 (-0.8--0.2) | 15 | 0.0077 - 0.048 | -0.69 - -0.56 |
| **ASSR Phase synchrony, a.u.** | 0.2±0.1 (0.2–0.3) | 0.3±0.13 (0.2-0.4) | 20 | 0.0013 - 0.046 | -0.73 - -0.54 |
