# Supplementary material for: Non-rapid eye movement sleep and wake neurophysiology in schizophrenia
Source: eLife. 2022 May 17;11:e76211. doi: 10.7554/eLife.76211 (PMC9113745; doi:10.7554/eLife.76211)
Supplement: Supplementary file 2. [file elife-76211-supp2.docx]

**Supplemental file 2**

**Non-rapid eye movement sleep and wake neurophysiology in schizophrenia**

**Authors:** Nataliia Kozhemiako^1†^, Jun Wang^2†^, Chenguang Jiang^2†^, Lei A. Wang^3^, Guan-chen Gai^2^, Kai Zou^2^, Zhe Wang^2^, Xiao-man Yu^2^, Lin Zhou^3^, Shen Li^4^, Zhenglin Guo^3^, Robert G. Law^1^, James Coleman^3^, Dimitrios Mylonas^5^, Lu Shen^7^, Guoqiang Wang^2^, Shuping Tan^6^, Shengying Qin^7^, Hailiang Huang^3,8^, Michael Murphy^4^, Robert Stickgold^9,10^, Dara S. Manoach^5^, Zhenhe Zhou^2•^, Wei Zhu^2•^, Mei-Hua Hall^4•^, Shaun M. Purcell^1,10•*^ & Jen Q. Pan^3•*^

**Affiliations:**

1. Department of Psychiatry, Brigham and Women’s Hospital, Harvard Medical School; Boston, USA
2. The Affiliated Wuxi Mental Health Center of Nanjing Medical University; Wuxi, China
3. Stanley Center for Psychiatric Research, Broad Institute of MIT and Harvard; Boston, USA
4. Department of Psychiatry, McLean Hospital, Harvard Medical School; Boston, USA
5. Department of Psychiatry, Massachusetts General Hospital, Harvard Medical School; Boston, USA
6. Huilong Guan Hospital, Beijing University; Beijing China
7. Bio-X Institutes, Shanghai Jiao Tong University; Shanghai China
8. ATGU, MGH, Harvard Medical School; Boston, USA
9. Beth Israel Deaconess Medical Center; Boston, USA
10. Department of Psychiatry, Harvard Medical School; Boston, USA

^†^ - co-first authors; • - co-senior authors

* - corresponding authors (Jen Q. Pan, jpan@broadinstitute.org ; Shaun M. Purcell, smpurcell@bwh.harvard.edu)

***Supplementary file 2a: Prescribed medications in the SCZ sample***

| **Medication** | **Number of subjects (total 72*)** |
| --- | --- |
| ***Antipsychotics*** | 69 |
| Amisulpride | 22 |
| Chlorpromazine Hydrochloride | 1 |
| Aripiprazole | 12 |
| Olanzapine | 26 |
| Clozapine | 12 |
| Quetiapine Fumarate | 6 |
| Risperidone | 14 |
| Paliperidone (sustained release) | 1 |
| ***Emotion stabilizer and Antiepileptic*** | 17 |
| ***Anticholinergics*** | 10 |
| ***Sedatives and Tranquilizers*** | 13 |
| ***Antidepressants*** | 4 |

*- due to 4 subjects being removed there were 67 out of 68. The table above shows the full dataset

***Supplementary file 2b: Medication associations with EEG metrics within the SCZ sample***

| **EEG metric** | **CPZ equivalent antipsychotic dose**  **(n=67)** | **Antipsychotic medication** | | | | | | **Adjunctive medication** | | |
| --- | --- | --- | --- | --- | --- | --- | --- | --- | --- | --- |
|  |  | **Amisulpride**  **(n=22)** | **Aripiprazole**  **(n=12)** | **Olanzapine**  **(n=26)** | **Clozapine**  **(n=12)** | **Quetiapine Fumarate**  **(n=6)** | **Risperidone**  **(n=14)** | **Sedatives and tranquilizers**  **(n=13)** | **Mood stabilizers and antiepileptics**  **(n=17)** | **Anticholinergics**  **(n=10)** |
| SS Density (↓ in SCZ) |  |  |  | ↑ FPZ,FP1,FP2 | ↓ AF3,F7,T7 |  |  |  |  |  |
| FS Density (↓ in SCZ) |  |  |  |  | ↓ 9 channels |  |  |  |  |  |
| SS Amplitude (↓ in SCZ) |  |  |  |  | ↓ AFZ,T7 |  |  |  |  | ↑ P2,P4 |
| FS Amplitude (↓ in SCZ) |  |  |  |  |  |  |  |  | ↓ AFZ |  |
| SS ISA (↓ in SCZ) |  |  | ↑ F2,F4 |  | ↓ T7 |  |  |  |  |  |
| FS ISA (↓ in SCZ) |  |  |  |  |  |  |  |  |  |  |
| FS Duration (↓ in SCZ) |  |  |  |  | ↓ 7 channels |  |  |  |  |  |
| FS Chirp (↓ in SCZ) | ↑FC6, ↓TP8 |  |  |  | ↓ 8 channels | ↓ FC1 |  |  |  |  |
| SO Density (↑ in SCZ) |  |  |  |  |  |  |  |  |  |  |
| SO Duration (↑ in SCZ) |  |  |  |  |  |  |  | ↑ 10 channels |  |  |
| SO Slope (↓ in SCZ) |  |  |  |  |  |  |  | ↓ 8 channels | ↓ 23 channels |  |
| SS overlap with SO (↓ in SCZ) |  |  | ↑ 6 channels |  | ↑ CP5,CZ,F5 |  |  |  | ↓ 6 channels |  |
| SO phase angle when SS occur (↓ in SCZ) |  |  | ↑ POZ |  |  |  |  |  | ↓ CPZ,CZ | ↑ C3,C4,C5 |
| SO phase angle when FS occur (↓ in SCZ) |  |  |  |  |  |  |  |  |  | ↑ CP3 |
| PSD PC #4 (↓ in SCZ) |  |  |  |  | ↓ |  |  |  |  |  |
| PSI PC #1 (↑ in SCZ) |  |  |  |  |  |  |  |  |  |  |
| MMN Amplitude (↓ in SCZ) |  |  | ↑ AF4 |  |  |  |  |  |  |  |
| P50 S2/S1 ratio (↑ in SCZ) |  |  |  |  |  |  |  |  |  |  |
| ASSR Power (↓ in SCZ) |  |  |  |  |  |  |  |  |  |  |
| ASSR Phase synchrony (↓ in SCZ) |  |  |  | ↑ 5 channels |  |  |  |  |  | ↓ F8 |

*A linear regression model with formula EEG metric ~ medication + sex + age + error was fit for each channel for each EEG metric, where medication was a vector with continuous data for Chlorpromazine (CPZ) equivalent antipsychotic dose and a binary vector (True or False for medication use) for other columns. Channels where the association was significant (unadjusted p<0.01) are provided in the table together with the direction of effect (↓ or ↑ in an EEG metric with medication)*
