# Supplementary material for: Non-rapid eye movement sleep and wake neurophysiology in schizophrenia
Source: eLife. 2022 May 17;11:e76211. doi: 10.7554/eLife.76211 (PMC9113745; doi:10.7554/eLife.76211)
Supplement: Supplementary file 3. [file elife-76211-supp3.docx]

**Supplemental file 3**

**Non-rapid eye movement sleep and wake neurophysiology in schizophrenia**

**Authors:** Nataliia Kozhemiako^1†^, Jun Wang^2†^, Chenguang Jiang^2†^, Lei A. Wang^3^, Guan-chen Gai^2^, Kai Zou^2^, Zhe Wang^2^, Xiao-man Yu^2^, Lin Zhou^3^, Shen Li^4^, Zhenglin Guo^3^, Robert G. Law^1^, James Coleman^3^, Dimitrios Mylonas^5^, Lu Shen^7^, Guoqiang Wang^2^, Shuping Tan^6^, Shengying Qin^7^, Hailiang Huang^3,8^, Michael Murphy^4^, Robert Stickgold^9,10^, Dara S. Manoach^5^, Zhenhe Zhou^2•^, Wei Zhu^2•^, Mei-Hua Hall^4•^, Shaun M. Purcell^1,10•*^ & Jen Q. Pan^3•*^

**Affiliations:**

1. Department of Psychiatry, Brigham and Women’s Hospital, Harvard Medical School; Boston, USA
2. The Affiliated Wuxi Mental Health Center of Nanjing Medical University; Wuxi, China
3. Stanley Center for Psychiatric Research, Broad Institute of MIT and Harvard; Boston, USA
4. Department of Psychiatry, McLean Hospital, Harvard Medical School; Boston, USA
5. Department of Psychiatry, Massachusetts General Hospital, Harvard Medical School; Boston, USA
6. Huilong Guan Hospital, Beijing University; Beijing China
7. Bio-X Institutes, Shanghai Jiao Tong University; Shanghai China
8. ATGU, MGH, Harvard Medical School; Boston, USA
9. Beth Israel Deaconess Medical Center; Boston, USA
10. Department of Psychiatry, Harvard Medical School; Boston, USA

^†^ - co-first authors; • - co-senior authors

* - corresponding authors (Jen Q. Pan, jpan@broadinstitute.org ; Shaun M. Purcell, smpurcell@bwh.harvard.edu)

***Supplementary file 3: Significance of group differences between SCZ and CTR adjusted for medication***

| **EEG metric** | **Antipsychotic medication** | | | | | | **Adjunctive medication** | | |
| --- | --- | --- | --- | --- | --- | --- | --- | --- | --- |
|  | **Amisulpride**  **(n=22)** | **Aripiprazole**  **(n=12)** | **Olanzapine**  **(n=26)** | **Clozapine**  **(n=12)** | **Quetiapine Fumarate**  **(n=6)** | **Risperidone**  **(n=14)** | **Sedatives and tranquilizers**  **(n=13)** | **Emotion stabilizers and antiepileptics**  **(n=17)** | **Anticholinergics**  **(n=10)** |
| SS Density, 20 channel(s) |  |  |  |  |  |  |  |  |  |
| FS Density, 53 channel(s) |  |  |  |  |  |  |  |  |  |
| SS Amplitude, 49 channel(s) |  |  |  |  |  |  |  |  |  |
| FS Amplitude, 22 channel(s) |  |  |  |  |  |  |  |  |  |
| SS ISA, 34 channel(s) |  |  |  |  |  |  |  |  |  |
| **FS ISA, 1 channel(s)** | n.s. |  | n.s. |  |  |  |  | n.s. |  |
| FS Duration, 35 channel(s) |  |  |  |  |  |  |  |  |  |
| FS Chirp, 20 channel(s) |  |  |  |  |  |  |  |  |  |
| SO Density, 30 channel(s) |  |  |  |  |  |  |  |  |  |
| SO Duration, 36 channel(s) |  |  |  |  |  |  |  |  |  |
| SO Slope, 44 channel(s) |  |  |  |  |  |  |  |  |  |
| SS overlap with SO, 13 channel(s) |  |  |  |  |  |  |  |  |  |
| SO phase angle when SS occur, 2 channel(s) |  |  |  |  |  |  |  |  |  |
| **SO phase angle when FS occur, 2 channel(s)** |  |  | n.s. |  |  |  |  | n.s. |  |
| PSD PC #4 |  |  |  |  |  |  |  |  |  |
| PSI PC #1 |  |  |  |  |  |  |  |  |  |
| **MMN Amplitude, 1 channel(s)** |  |  | n.s. |  |  |  |  |  |  |
| P50 S2/S1 ratio, 10 channel(s) |  |  |  |  |  |  |  |  |  |
| ASSR Power, 15 channel(s) |  |  |  |  |  |  |  |  |  |
| ASSR Phase synchrony, 20 channel(s) |  |  |  |  |  |  |  |  |  |

*For each medication specified in the columns, SCZ patients taking that medication were excluded from the SCZ sample and group differences for each EEG metric at each channel were re-estimated.* ***n.s.*** *indicates that there were no channels with significant differences (unadjusted p<0.01) once subjects taking the corresponding medication were excluded.*
