# Supplementary material for: Non-rapid eye movement sleep and wake neurophysiology in schizophrenia
Source: eLife. 2022 May 17;11:e76211. doi: 10.7554/eLife.76211 (PMC9113745; doi:10.7554/eLife.76211)
Supplement: Supplementary file 4. [file elife-76211-supp4.docx]

**Supplemental file 4**

**Non-rapid eye movement sleep and wake neurophysiology in schizophrenia**

**Authors:** Nataliia Kozhemiako^1†^, Jun Wang^2†^, Chenguang Jiang^2†^, Lei A. Wang^3^, Guan-chen Gai^2^, Kai Zou^2^, Zhe Wang^2^, Xiao-man Yu^2^, Lin Zhou^3^, Shen Li^4^, Zhenglin Guo^3^, Robert G. Law^1^, James Coleman^3^, Dimitrios Mylonas^5^, Lu Shen^7^, Guoqiang Wang^2^, Shuping Tan^6^, Shengying Qin^7^, Hailiang Huang^3,8^, Michael Murphy^4^, Robert Stickgold^9,10^, Dara S. Manoach^5^, Zhenhe Zhou^2•^, Wei Zhu^2•^, Mei-Hua Hall^4•^, Shaun M. Purcell^1,10•*^ & Jen Q. Pan^3•*^

**Affiliations:**

1. Department of Psychiatry, Brigham and Women’s Hospital, Harvard Medical School; Boston, USA
2. The Affiliated Wuxi Mental Health Center of Nanjing Medical University; Wuxi, China
3. Stanley Center for Psychiatric Research, Broad Institute of MIT and Harvard; Boston, USA
4. Department of Psychiatry, McLean Hospital, Harvard Medical School; Boston, USA
5. Department of Psychiatry, Massachusetts General Hospital, Harvard Medical School; Boston, USA
6. Huilong Guan Hospital, Beijing University; Beijing China
7. Bio-X Institutes, Shanghai Jiao Tong University; Shanghai China
8. ATGU, MGH, Harvard Medical School; Boston, USA
9. Beth Israel Deaconess Medical Center; Boston, USA
10. Department of Psychiatry, Harvard Medical School; Boston, USA

^†^ - co-first authors; • - co-senior authors

* - corresponding authors (Jen Q. Pan, jpan@broadinstitute.org ; Shaun M. Purcell, smpurcell@bwh.harvard.edu)

***Supplementary file 4 Association between clinical variables and EEG metrics in the SCZ sample***

| **EEG metric** | **SCZ Duration** | **PANSS 5 factors** | | | | |
| --- | --- | --- | --- | --- | --- | --- |
|  |  | **Positive** | **Negative** | **Disorganized Concrete** | **Excited** | **Depressed** |
| SS Density (↓ in SCZ) |  |  | ↓ AF3,AF4,F8 | ↓ 24 channels |  |  |
| FS Density (↓ in SCZ) |  |  |  |  |  |  |
| SS Amplitude (↓ in SCZ) |  |  | ↓ 15 channels |  |  |  |
| FS Amplitude (↓ in SCZ) |  |  | ↓ 5 channels |  |  |  |
| SS ISA (↓ in SCZ) |  |  |  |  |  |  |
| FS ISA (↓ in SCZ) |  | ↑ F6,F7,F8,FC6 |  |  |  |  |
| FS Duration (↓ in SCZ) |  |  |  |  |  |  |
| FS Chirp (↓ in SCZ) |  |  |  |  |  |  |
| SO Density (↑ in SCZ) | ↓ 17 channels |  |  |  |  |  |
| SO Duration (↑ in SCZ) |  |  | ↓ F6 |  |  |  |
| SO Slope (↓ in SCZ) |  |  |  |  | ↑ FT8,T8,TP7 |  |
| SS overlap with SO (↓ in SCZ) |  |  |  |  |  |  |
| SO phase angle when SS occur  (↓ in SCZ) | ↑ FPZ,FP2 |  |  | ↓ FCZ | ↓ FC2,FCZ |  |
| SO phase angle when FS occur  (↓ in SCZ) | ↓ FT8,O1,P8,TP8 |  |  |  |  |  |
| PSD PC #4 (↓ in SCZ) |  |  |  |  |  |  |
| PSI PC #1 (↑ in SCZ) |  |  |  |  |  |  |
| MMN Amplitude (↓ in SCZ) |  |  | ↓ 7 channels | ↓ FT7,T7 |  |  |
| P50 S2/S1 ratio (↑ in SCZ) |  | ↑ FZ | ↑ C1,CZ |  | ↑ F1 |  |
| ASSR Power (↓ in SCZ) | ↓ O1 |  |  |  |  |  |
| ASSR Phase synchrony (↓ in SCZ) |  |  |  |  |  |  |

*A linear regression model with formula EEG metric ~ clinical variable + sex + age + error was fit for each channel / EEG metric. Channels with significant association (unadjusted p<0.01) are listed together with the direction of effect (↓ or ↑ in an EEG metric with clinical variable)*
