# Supplementary material for: Non-rapid eye movement sleep and wake neurophysiology in schizophrenia
Source: eLife. 2022 May 17;11:e76211. doi: 10.7554/eLife.76211 (PMC9113745; doi:10.7554/eLife.76211)
Supplement: Supplementary file 5. [file elife-76211-supp5.docx]

**Supplemental file 5**

**Non-rapid eye movement sleep and wake neurophysiology in schizophrenia**

**Authors:** Nataliia Kozhemiako^1†^, Jun Wang^2†^, Chenguang Jiang^2†^, Lei A. Wang^3^, Guan-chen Gai^2^, Kai Zou^2^, Zhe Wang^2^, Xiao-man Yu^2^, Lin Zhou^3^, Shen Li^4^, Zhenglin Guo^3^, Robert G. Law^1^, James Coleman^3^, Dimitrios Mylonas^5^, Lu Shen^7^, Guoqiang Wang^2^, Shuping Tan^6^, Shengying Qin^7^, Hailiang Huang^3,8^, Michael Murphy^4^, Robert Stickgold^9,10^, Dara S. Manoach^5^, Zhenhe Zhou^2•^, Wei Zhu^2•^, Mei-Hua Hall^4•^, Shaun M. Purcell^1,10•*^ & Jen Q. Pan^3•*^

**Affiliations:**

1. Department of Psychiatry, Brigham and Women’s Hospital, Harvard Medical School; Boston, USA
2. The Affiliated Wuxi Mental Health Center of Nanjing Medical University; Wuxi, China
3. Stanley Center for Psychiatric Research, Broad Institute of MIT and Harvard; Boston, USA
4. Department of Psychiatry, McLean Hospital, Harvard Medical School; Boston, USA
5. Department of Psychiatry, Massachusetts General Hospital, Harvard Medical School; Boston, USA
6. Huilong Guan Hospital, Beijing University; Beijing China
7. Bio-X Institutes, Shanghai Jiao Tong University; Shanghai China
8. ATGU, MGH, Harvard Medical School; Boston, USA
9. Beth Israel Deaconess Medical Center; Boston, USA
10. Department of Psychiatry, Harvard Medical School; Boston, USA

^†^ - co-first authors; • - co-senior authors

* - corresponding authors (Jen Q. Pan, jpan@broadinstitute.org ; Shaun M. Purcell, smpurcell@bwh.harvard.edu)

***Supplementary file 5 Demographic characteristics of the independent samples***

| **Sample characteristics** | **Lunesta dataset**  (4-7 EEG channels:  F3, F4, C3,C4,Pz,O1,O2) | | **ESZ dataset**  (58 EEG channels) | | **GCRC dataset**  (4 EEG channels: C3,C4,O1,O2) | |
| --- | --- | --- | --- | --- | --- | --- |
|  | **SCZ** | **CTR** | **SCZ** | **CTR** | **SCZ** | **CTR** |
| N | 20 | 17 | 26 | 29 | 11 | 13 |
| Sex | 5 females | 3 females | 5 females | 8 females | 3 females | 3 females |
| Race | White - 15; Black - 3;  Asian - 1; | White - 17 | White - 12; Black - 6;  Asian - 3; na - 5 | White - 17; Asian - 3; na - 9 | White - 9; Black - 1;  Native American - 1; | White - 11; Black - 1;  Multiracial - 1; |
| Age, years | 34.9±8.69 | 36.3±7.12 | 32.3±7.53 | 30.1±6.25 | 44.1±9.5 | 43.1±6.11 |
| Parental education, years | 15.1±3.33 | 13.5±2.15 | 13.4±3.01 | **16.7±2.02^*^** | 13.1±1.73 | 13.5±3.02 |
| Std. antipsychotic dose,  mg | 314±240.15 |  | 416.5±310 |  | 567.2±512.87 |  |
| **Sleep macrostructure parameters** | | | | | |  |
| Total time in bed, mins | **622±75*** | 578±44 | 575±42.2 | 561±42.5 | **610±70*** | 563±50.1 |
| Total sleep time, mins | **464±72.6*** | 420±52.4 | 510±71.1 | 506±45.7 | 430±101.7 | 390±71 |
| Sleep latency, mins | **19±13.7*** | 8±4.9 | 26±17.5 | 15±10.2 | 106±66.7 | 113±30.3 |
| Wake after sleep onset,  mins | 33±33.1 | 35±17.5 | **22±16.8*** | 33±19.2 | 62±55.7 | 47±23.3 |
| Sleep efficiency (/TIB) | 87±8.6 | 88±4.9 | 90±6.6 | 91±3.6 | 71±14.2 | 69±8 |
| Sleep efficiency  (/start-end of sleep) | 92±8.6 | 92±3.4 | **96±3*** | 94±3.5 | 86±10.9 | 88±5.7 |
| Duration N1, mins (%) | 33±22.7 (8) | 37±16.1 (9) | 44±22.4 (9) | 52±22 (10) | 32±18.5 (8) | 24±13.3 (6) |
| Duration N2, mins (%) | 233±68.7 (53) | 234±36.5 (56) | 266±79 (53) | 265±54.4 (52) | 297±94.6 (69) | 240±66.6 (64) |
| Duration N3, mins (%) | 83±50.4 (18) | 56±23 (13) | 93±55.3 (19) | 93±32 (19) | 44±39.5 (10) | 42±20.8 (11) |
| Duration REM, mins (%) | 97±38.2 (22) | 92±22 (22) | 88±29.7 (17) | 92±29.6 (18) | 57±31.1 (13) | 84±42.9 (21) |
| Latency of REM, mins | 150±84.9 | 100±49.1 | 123±59.6 | 106±40.2 | **195±72.6*** | 114±76.4 |
| Number of cycles | 4±1.2 | 5±1.2 | 5±1.4 | 5±1 | 4±1.2 | 4±1.2 |
| Cycle length, mins | **116±34.4*** | 94±19.4 | 112±23.4 | 97±15.1 | 115±38.7 | 96±21.1 |

** p-value<0.05*
