# Supplementary material for: Non-rapid eye movement sleep and wake neurophysiology in schizophrenia
Source: eLife. 2022 May 17;11:e76211. doi: 10.7554/eLife.76211 (PMC9113745; doi:10.7554/eLife.76211)
Supplement: Supplementary file 6. [file elife-76211-supp6.docx]

**Supplemental file 6**

**Non-rapid eye movement sleep and wake neurophysiology in schizophrenia**

**Authors:** Nataliia Kozhemiako^1†^, Jun Wang^2†^, Chenguang Jiang^2†^, Lei A. Wang^3^, Guan-chen Gai^2^, Kai Zou^2^, Zhe Wang^2^, Xiao-man Yu^2^, Lin Zhou^3^, Shen Li^4^, Zhenglin Guo^3^, Robert G. Law^1^, James Coleman^3^, Dimitrios Mylonas^5^, Lu Shen^7^, Guoqiang Wang^2^, Shuping Tan^6^, Shengying Qin^7^, Hailiang Huang^3,8^, Michael Murphy^4^, Robert Stickgold^9,10^, Dara S. Manoach^5^, Zhenhe Zhou^2•^, Wei Zhu^2•^, Mei-Hua Hall^4•^, Shaun M. Purcell^1,10•*^ & Jen Q. Pan^3•*^

**Affiliations:**

1. Department of Psychiatry, Brigham and Women’s Hospital, Harvard Medical School; Boston, USA
2. The Affiliated Wuxi Mental Health Center of Nanjing Medical University; Wuxi, China
3. Stanley Center for Psychiatric Research, Broad Institute of MIT and Harvard; Boston, USA
4. Department of Psychiatry, McLean Hospital, Harvard Medical School; Boston, USA
5. Department of Psychiatry, Massachusetts General Hospital, Harvard Medical School; Boston, USA
6. Huilong Guan Hospital, Beijing University; Beijing China
7. Bio-X Institutes, Shanghai Jiao Tong University; Shanghai China
8. ATGU, MGH, Harvard Medical School; Boston, USA
9. Beth Israel Deaconess Medical Center; Boston, USA
10. Department of Psychiatry, Harvard Medical School; Boston, USA

^†^ - co-first authors; • - co-senior authors

* - corresponding authors (Jen Q. Pan, jpan@broadinstitute.org ; Shaun M. Purcell, smpurcell@bwh.harvard.edu)

***Supplementary file 6 Replication analysis for spindle, slow oscillation and coupling metrics***

| **Sleep microstructure metrics** | **EEG channel** | **GRINS** | | | | **Replication** | | | |
| --- | --- | --- | --- | --- | --- | --- | --- | --- | --- |
|  |  | **SCZ**  **Mean ± SD** | **CTR**  **Mean ± SD** | **p-value** | **Effect size., SD** | **SCZ**  **Mean ± SD** | **CTR**  **Mean ± SD** | **p-value** | **Effect size SD** |
| **SS density, n/min** | C3 | 1.2±0.45 | 1.4±0.4 | **0.0179** | -0.45 | 1±0.43 | 1.2±0.47 | **0.0053** | -0.51 |
|  | C4 | 1.1±0.47 | 1.3±0.42 | **0.0312** | -0.45 | 0.9±0.43 | 1.2±0.47 | **0.004** | -0.58 |
|  | O1 | 0.7±0.35 | 0.9±0.35 | **7E-04** | -0.66 | 0.6±0.3 | 0.7±0.33 | **0.0139** | -0.46 |
|  | O2 | 0.8±0.38 | 1±0.32 | **0.0079** | -0.59 | 0.5±0.28 | 0.7±0.31 | **0.0012** | -0.61 |
| **FS density, n/min** | C3 | 1.9±0.9 | 2.7±0.78 | **0** | -1.03 | 1.7±0.94 | 2.6±0.76 | **0** | -1.14 |
|  | C4 | 2±0.86 | 2.8±0.75 | **0** | -1.14 | 1.6±0.93 | 2.3±0.84 | **0** | -0.91 |
|  | O1 | 1.8±0.99 | 2.4±1.04 | **0.0011** | -0.59 | 1.2±0.95 | 1.8±1.02 | **9E-04** | -0.61 |
|  | O2 | 1.8±0.98 | 2.5±1.07 | **9E-04** | -0.63 | 1.2±0.97 | 1.8±0.94 | **9E-04** | -0.61 |
| SS amplitude, uV | C3 | 24.9±6.46 | 29.5±5.99 | **0** | -0.78 | 24.1±5.95 | 25.5±7.66 | 0.3713 | -0.19 |
|  | C4 | 25.9±6.7 | 31.3±7.22 | **1E-04** | -0.75 | 25±6.6 | 25.4±6.1 | 0.7147 | -0.06 |
|  | O1 | 18.2±5.75 | 21.2±6.08 | **0.0064** | -0.49 | 16.9±4.66 | 18±6.59 | 0.4946 | -0.17 |
|  | O2 | 18.6±5.78 | 21.8±6.37 | **0.0026** | -0.51 | 17.4±5.08 | 18.3±6.05 | 0.3522 | -0.14 |
| **FS amplitude, uV** | C3 | 18.5±4.59 | 21.6±5.54 | **0.0021** | -0.57 | 16.7±4.42 | 19.6±6.08 | **0.017** | -0.48 |
|  | C4 | 19.7±5.14 | 22.6±5.27 | **0.0015** | -0.55 | 17.2±4.62 | 19±4.77 | 0.0782 | -0.37 |
|  | O1 | 14.2±4.95 | 15.3±5.26 | 0.1123 | -0.22 | 11.5±3.34 | 13±4.91 | 0.1274 | -0.29 |
|  | O2 | 14.5±4.73 | 16.3±5.99 | **0.0389** | -0.3 | 11.7±3.75 | 13.1±4.56 | 0.1051 | -0.31 |
| **SS ISA, a.u.** | C3 | 1.3±0.2 | 1.4±0.2 | **4E-04** | -0.9 | 1.2±0.19 | 1.4±0.17 | **1E-04** | -0.92 |
|  | C4 | 1.3±0.22 | 1.4±0.19 | **0.0012** | -0.82 | 1.2±0.2 | 1.3±0.18 | **0** | -0.86 |
|  | O1 | 1.3±0.31 | 1.4±0.24 | **0.0303** | -0.65 | 1.2±0.25 | 1.4±0.27 | **1E-04** | -0.72 |
|  | O2 | 1.3±0.28 | 1.4±0.19 | **0.0049** | -0.83 | 1.2±0.3 | 1.4±0.28 | **0.0016** | -0.57 |
| **FS ISA, a.u.** | C3 | 1.3±0.18 | 1.4±0.13 | 0.084 | -0.48 | 1.3±0.21 | 1.4±0.15 | **0.0287** | -0.49 |
|  | C4 | 1.3±0.18 | 1.4±0.16 | **0.0492** | -0.54 | 1.3±0.21 | 1.4±0.16 | 0.0746 | -0.42 |
|  | O1 | 1.3±0.2 | 1.4±0.15 | 0.1626 | -0.37 | 1.2±0.21 | 1.3±0.19 | **0.0027** | -0.58 |
|  | O2 | 1.4±0.18 | 1.4±0.15 | 0.0784 | -0.41 | 1.2±0.2 | 1.3±0.18 | **0.0023** | -0.56 |
| **FS duration, s** | C3 | 0.8±0.09 | 0.8±0.07 | **0.0032** | -0.79 | 0.8±0.12 | 0.8±0.09 | **0.0459** | -0.45 |
|  | C4 | 0.8±0.09 | 0.8±0.07 | **0.0031** | -0.82 | 0.8±0.11 | 0.8±0.09 | 0.0596 | -0.41 |
|  | O1 | 0.8±0.11 | 0.9±0.07 | **0.0056** | -0.85 | 0.8±0.11 | 0.8±0.09 | **0.0134** | -0.62 |
|  | O2 | 0.8±0.11 | 0.9±0.08 | **0.0119** | -0.7 | 0.8±0.12 | 0.8±0.09 | **0.0072** | -0.55 |
| **FS chirp, Hz** | C3 | -0.2±0.09 | -0.2±0.1 | 0.1766 | -0.3 | -0.2±0.09 | -0.2±0.1 | 0.1655 | -0.26 |
|  | C4 | -0.2±0.1 | -0.2±0.1 | **0.0177** | -0.47 | -0.2±0.1 | -0.2±0.09 | 0.5148 | -0.07 |
|  | O1 | -0.2±0.11 | -0.1±0.08 | **6E-04** | -1.09 | -0.1±0.1 | -0.1±0.07 | **0.0379** | -0.51 |
|  | O2 | -0.2±0.1 | -0.1±0.08 | **4E-04** | -0.94 | -0.1±0.08 | -0.1±0.07 | 0.0854 | -0.33 |
| SO density, n/min | C3 | 13.4±2.08 | 12.5±1.59 | **0.0144** | 0.57 | 12.9±1.99 | 12.3±1.28 | 0.3876 | 0.44 |
|  | C4 | 13.3±2.1 | 12.5±1.58 | **0.023** | 0.52 | 13±2.07 | 12.1±1.29 | 0.0615 | 0.72 |
|  | O1 | 12.1±2.16 | 10.2±1.61 | **0** | 1.16 | 11.2±1.59 | 10.2±1.43 | **0.0033** | 0.67 |
|  | O2 | 11.9±2.13 | 10.2±1.63 | **0** | 1.06 | 11±1.64 | 10.2±1.28 | **0.0108** | 0.61 |
| SO duration, s | C3 | 1±0.18 | 0.9±0.12 | **2E-04** | 1.08 | 1±0.12 | 0.9±0.1 | 0.0884 | 0.52 |
|  | C4 | 1±0.19 | 0.9±0.09 | **0** | 1.67 | 0.9±0.11 | 0.9±0.12 | 0.2306 | 0.22 |
|  | O1 | 1.3±0.32 | 1.1±0.2 | **0.0022** | 0.93 | 1.1±0.22 | 1±0.17 | 0.6103 | 0.32 |
|  | O2 | 1.3±0.32 | 1.1±0.18 | **0.0013** | 1.16 | 1.1±0.18 | 1±0.15 | 0.2733 | 0.46 |
| SO slope, a.u. | C3 | 181.2±41.52 | 222.8±45.87 | **0** | -0.91 | 194.7±43.3 | 198.8±38.16 | 0.7308 | -0.11 |
|  | C4 | 188±43.82 | 236.7±48.31 | **0** | -1.01 | 194.2±44.34 | 197±33.89 | 0.9467 | -0.08 |
|  | O1 | 107.9±32.63 | 122.7±33.92 | **0.0451** | -0.44 | 108.2±28.98 | 108±29.97 | 0.7957 | 0 |
|  | O2 | 110.4±31.75 | 134.3±41.45 | **0.0027** | -0.58 | 108.5±29.44 | 110.4±29.13 | 0.999 | -0.07 |
| SS SO coupling overlap, z | C3 | 2.3±0.94 | 2.8±0.91 | **0.0065** | -0.57 | 2.6±1.2 | 2.9±0.73 | 0.2632 | -0.53 |
|  | C4 | 2.1±1.05 | 2.8±0.9 | **4E-04** | -0.79 | 2.2±1.61 | 2.9±0.69 | 0.0872 | -1.06 |
|  | O1 | 1.3±1.24 | 2±1.06 | **0.0018** | -0.75 | 1.1±1.34 | 2±1.1 | **0.0016** | -0.83 |
|  | O2 | 1.3±1.33 | 1.9±1.09 | **0.002** | -0.63 | 1.1±1.39 | 1.9±1.21 | **0.0048** | -0.68 |
| SO phase angle at SS peak,° | C3 | 356±57.8 | 379.7±48.8 | 0.0776 | -0.49 | 386.7±56.66 | 382.7±53.21 | 0.6712 | 0.07 |
|  | C4 | 361.9±57.73 | 383.8±47.97 | **0.026** | -0.46 | 380.4±51.89 | 389.1±45.66 | 0.3559 | -0.19 |
|  | O1 | 359.1±108.55 | 384.9±85.24 | 0.3365 | -0.3 | 376±88.78 | 385.9±89.2 | 0.7236 | -0.11 |
|  | O2 | 338.2±116.09 | 368.5±93.09 | 0.0595 | -0.33 | **360.2±100.05** | **414.1±87.04** | **0.0088** | **-0.62** |
| SO phase angle at FS peak,° | C3 | 239.2±15.03 | 240.3±22.24 | 0.8406 | -0.05 | 267.9±27.54 | 266.6±26.47 | 0.95 | 0.05 |
|  | C4 | 237.3±14.54 | 243±17.51 | 0.1556 | -0.32 | 269.3±31.25 | 262.4±33.87 | 0.7349 | 0.2 |
|  | O1 | 218.8±26.06 | 225.9±38.68 | 0.2162 | -0.18 | 261.3±29.21 | 253.6±38.16 | 0.6079 | 0.2 |
|  | O2 | 222.1±29.36 | 220.6±52.05 | 0.5997 | 0.03 | 257.1±31.71 | 253.3±34.33 | 0.7406 | 0.11 |
| SS phase-frequency coupling, a.u. | C3 | 0.7±0.22 | 0.8±0.17 | 0.0095 | -0.74 | 0.6±0.3 | 0.7±0.2 | 0.0514 | -0.57 |
|  | C4 | 0.6±0.26 | 0.8±0.18 | 0.0016 | -0.9 | 0.7±0.21 | 0.7±0.24 | 0.5336 | -0.17 |
|  | O1 | 0.5±0.24 | 0.5±0.26 | 0.2754 | -0.21 | 0.4±0.3 | 0.5±0.27 | 0.386 | -0.21 |
|  | O2 | 0.5±0.26 | 0.6±0.27 | 0.1129 | -0.4 | 0.4±0.31 | 0.5±0.26 | 0.0643 | -0.46 |
