# Supplementary material for: Non-rapid eye movement sleep and wake neurophysiology in schizophrenia
Source: eLife. 2022 May 17;11:e76211. doi: 10.7554/eLife.76211 (PMC9113745; doi:10.7554/eLife.76211)
Supplement: Transparent reporting form [file elife-76211-transrepform1.docx]

***eLife’s* transparent reporting form**

We encourage authors to provide detailed information *within their submission* to facilitate the interpretation and replication of experiments. Authors can upload supporting documentation to indicate the use of appropriate reporting guidelines for health-related research (see [EQUATOR Network](http://www.equator-network.org/%20)), life science research (see the [BioSharing Information Resource](https://biosharing.org/)), or the [ARRIVE guidelines](http://www.plosbiology.org/article/info:doi/10.1371/journal.pbio.1000412) for reporting work involving animal research. Where applicable, authors should refer to any relevant reporting standards documents in this form.

If you have any questions, please consult our Journal Policies and/or contact us: [editorial@elifesciences.org](mailto:editorial@elifesciences.org).

**Sample-size estimation**

- You should state whether an appropriate sample size was computed when the study was being designed
- You should state the statistical method of sample size computation and any required assumptions
- If no explicit power analysis was used, you should describe how you decided what sample (replicate) size (number) to use

Please outline where this information can be found within the submission (e.g., sections or figure legends), or explain why this information doesn’t apply to your submission:

In this study, we aimed to obtain one of the largest samples up to date to investigate multiple parameters of sleep EEG. Primarily orienting on sleep spindle findings, we used a recent report (Mylonas et.al. 2020) to obtain an effect size and a group standard deviation for spindle density deficit in patients with schizophrenia. Our sample size calculations were based on guidelines outlined in Kadam et al. 2010 using next parameters to ensure low probabilities of both type I and type II errors (two-sided t-test at the 99% confidence level (α=0.01) and 95% power). According to those calculations, the required sample to detect such effect would be 123 individuals.

**Replicates**

- You should report how often each experiment was performed
- You should include a definition of biological versus technical replication
- The data obtained should be provided and sufficient information should be provided to indicate the number of independent biological and/or technical replicates
- If you encountered any outliers, you should describe how these were handled
- Criteria for exclusion/inclusion of data should be clearly stated
- High-throughput sequence data should be uploaded before submission, with a private link for reviewers provided (these are available from both GEO and ArrayExpress)

Please outline where this information can be found within the submission (e.g., sections or figure legends), or explain why this information doesn’t apply to your submission:

In addition to the main analysis, we used a demographically distinct cohort of comparable sample size to replicate our main sleep EEG findings in this manuscript. The details are reported in the *Independent replication of NREM sleep metric SCZ associations* subsection of results*, Replication datasets* subsection and *Statistical Analysis* subsection of the Methods and Materials section.

The information on outlier detection and exclusion of individuals from some analyses due to poor data quality, EEG polarity issues or insufficient number of channels can be found in *Staging and Pre-processing*, *Replication datasets* and *Statistical Analysis* subsections.

**Statistical reporting**

- Statistical analysis methods should be described and justified
- Raw data should be presented in figures whenever informative to do so (typically when N per group is less than 10)
- For each experiment, you should identify the statistical tests used, exact values of N, definitions of center, methods of multiple test correction, and dispersion and precision measures (e.g., mean, median, SD, SEM, confidence intervals; and, for the major substantive results, a measure of effect size (e.g., Pearson's r, Cohen's d)
- Report exact p-values wherever possible alongside the summary statistics and 95% confidence intervals. These should be reported for all key questions and not only when the p-value is less than 0.05.

Please outline where this information can be found within the submission (e.g., sections or figure legends), or explain why this information doesn’t apply to your submission:

Description of all statistical analyses is provided in *Statistical Analysis* subsection. This subsection also contains a description of how the results were corrected for multiple comparisons. The figures reporting main results (Fig. 1-3, 5, 8-9) contain the raw data plots at an exemplary channel for the metrics with significant group differences. Each of those highlight such information as a mean, SD, outliers, and a p-value (adjusted for multiple comparisons) and effect size. In addition, the results of statistical comparisons across all tested EEG metrics for the main analyses are summarized in the Supplementary table 1 and for the replication analyses in the Supplementary table 7.

(For large datasets, or papers with a very large number of statistical tests, you may upload a single table file with tests, Ns, etc., with reference to sections in the manuscript.)

**Group allocation**

- Indicate how samples were allocated into experimental groups (in the case of clinical studies, please specify allocation to treatment method); if randomization was used, please also state if restricted randomization was applied
- Indicate if masking was used during group allocation, data collection and/or data analysis

Please outline where this information can be found within the submission (e.g., sections or figure legends), or explain why this information doesn’t apply to your submission:

Recruitment information of patients and control subject were detailed in the *Participant cohort* subsection. The same subsection also contains information on inclusion and exclusion criteria for patients with schizophrenia and control participants.

**Additional data files (“source data”)**

- We encourage you to upload relevant additional data files, such as numerical data that are represented as a graph in a figure, or as a summary table
- Where provided, these should be in the most useful format, and they can be uploaded as “Source data” files linked to a main figure or table
- Include model definition files including the full list of parameters used
- Include code used for data analysis (e.g., R, MatLab)
- Avoid stating that data files are “available upon request”

Please indicate the figures or tables for which source data files have been provided:

Anonymized individual-level data for the derived EEG metrics, disease status, and demographics are available on the Dryad archive (**doi:10.5061/dryad.j0zpc86h4**). R scripts to reproduce the key figures are available upon request.
